# Supplementary material for: Virucidal and Bactericidal Filtration Media from Electrospun Polylactic Acid Nanofibres Capable of Protecting against COVID-19
Source: Membranes (Basel). 2022 May 30;12(6):571. doi: 10.3390/membranes12060571 (PMC9227935; doi:10.3390/membranes12060571)
Supplement: Supplementary file 1 [file membranes-12-00571-s001.zip › membranes-1714287-supplementary.pdf]

membranes

Supplementary Material

Virucidal and Bactericidal Filtration Media from Electrospun Polylactic Acid Nanofibre Capable of Protecting Against COVID-19

Fabrice N. H. Karabulut [1,*], Dhevesh Fomra [1,*], Günther Höfler [1], Naveen A. Chand [1] and Gareth W. Beckermann [1]

[1] NanoLayr Ltd., 59 Mahunga Drive, Mangere Bridge, Auckland 2022, New Zealand
* Correspondence: fabrice.karabulut@nanolayr.com; dhevesh.fomra@nanolayr.com

2.6. Antimicrobial activity

2.6.1. Antibacterial activity

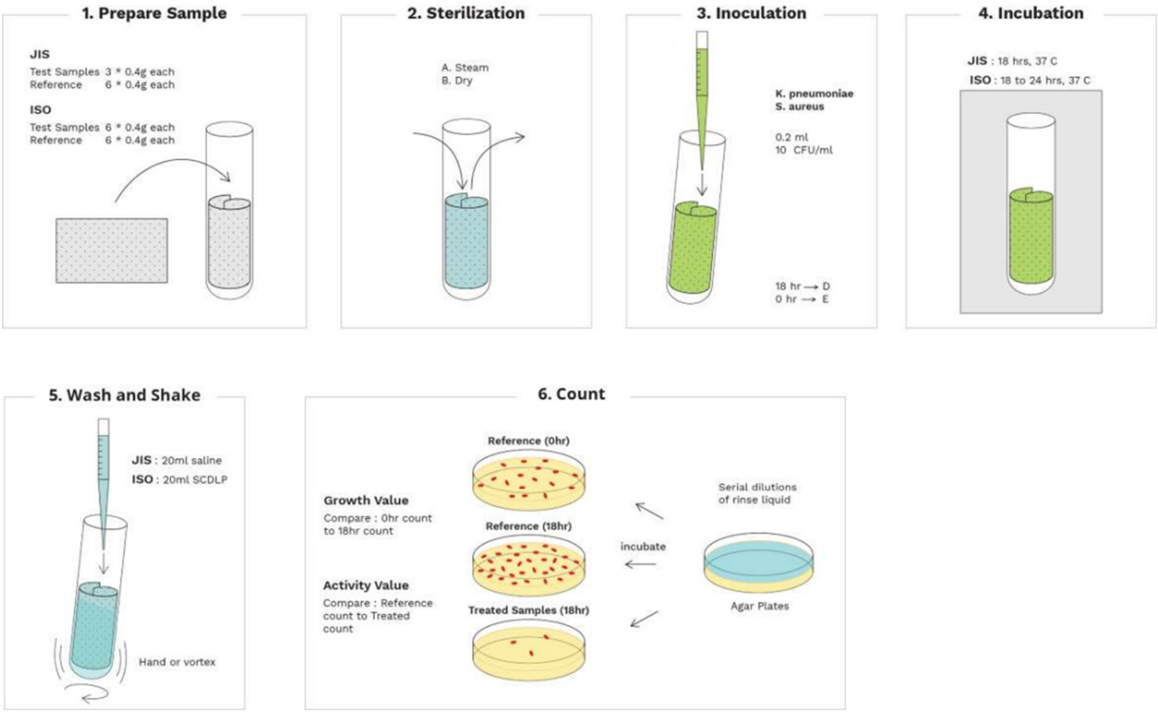

Figure S1. Illustration of the Antibacterial testing procedure.

2.6.2. Antiviral activity

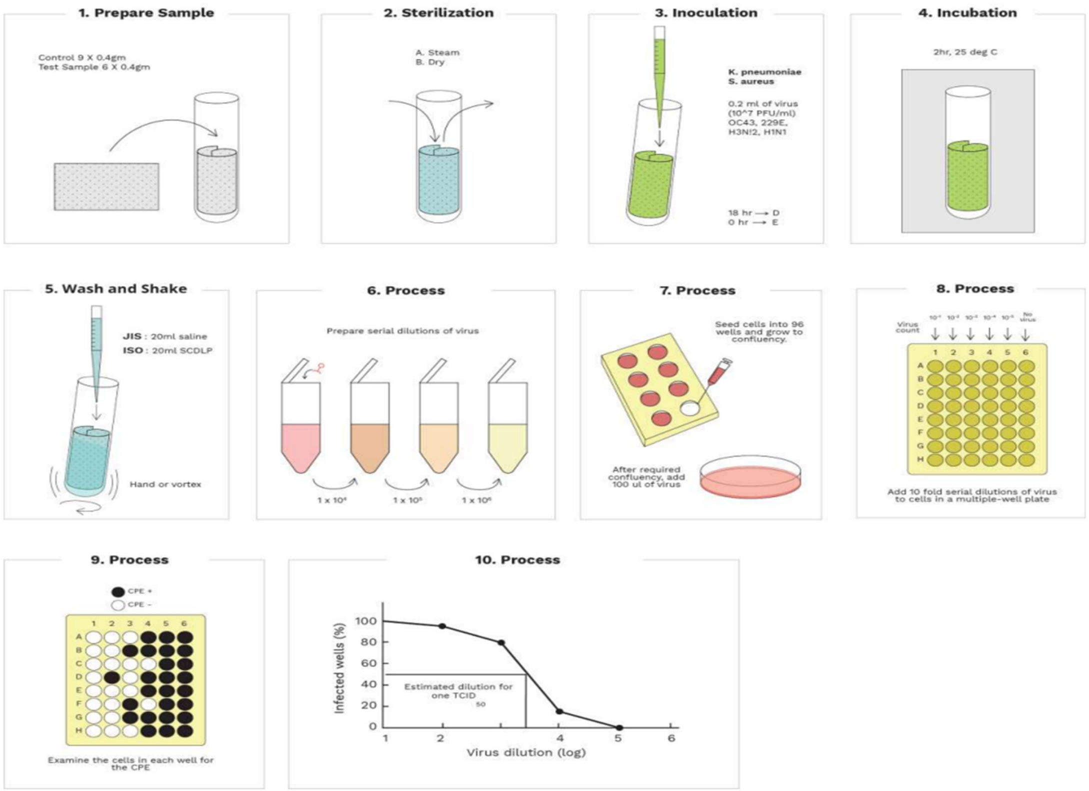

Figure S2. Illustration of the Antiviral testing procedure.

3.1. Filtration performance of PLA nanofibers containing Manuka triketone against international standard: ASTM F2299, ASTM F3502 and NIOSH 42CFR84

3.1.1. ASTM F2100 - Standard Specification for Performance of Materials Used in Medical Face Masks

Table S1. Filtration efficiency of PLA NF filter when challenge with 0.1 µm PSL particles following ASTM F2299 test method, certified by Nelson Lab.

Test Article Number	Test Article Counts	Average Control Counts	0.1 µm Filtration Efficiency (%)
1	22	13,160	99.83
2	14	13,059	99.89
3	6	13,031	99.95
4	23	12,940	99.82
5	21	12,717	99.83
ASTM F2100 Requirements for Level 2 pass	-	-	≥98

Table S2. Particles filtration efficiency of PLA NF filter when challenge with 0.3 µm PSL particles following ASTM F2299 test method, certified by Nelson Lab.

Test Article Number	Test Article Counts	Average Control Counts	0.3 µm Filtration Efficiency (%)
1	9	12,213	99.926
2	10	12,354	99.919
3	7	12,431	99.944
4	10	12,615	99.921
5	11	12,454	99.912

6	8	12,829	99.938
7	9	12,947	99.93
8	3	12,637	99.976
9	6	12,433	99.952
10	3	12,443	99.976
11	2	12,157	99.984
12	4	12,436	99.968
13	9	12,544	99.928
14	6	12,494	99.952
15	15	12,755	99.88
16	9	12,905	99.93
17	8	12,944	99.938
18	4	12,976	99.969
19	6	13,036	99.954
20	8	13,132	99.939

Conclusions: The filter media exceeded the ASTM F2100 Level 2 particle filtration efficiency requirements of ≥98% for 0.1 μm sized particles and showed >99.9% filtration efficiency for 0.3 μm sized particles.

Table S3. Pressure drops values of PLA NF filter when tested against EN 14683:2019+AC:2019 test method, certified by Nelson Lab.

Test Article Number	Delta P (mm H2O/cm2)	Delta P (Pa/cm2)
1	4.7	46
2	4.6	45
3	4.4	43
ASTM F2100 Requirements for Level 2 pass	<6.0	<58.8

Differential Pressure: The Delta P test is performed to determine the breathability of test articles by measuring the differential air pressure on either side of the test article using a manometer, at a constant flow rate. The Delta P test complies with EN 14683:2019+AC:2019.

Table S4. Bacterial filtration efficiency of PLA NF filter media.

Test Article Number	Percent BFE (%)
1	>99.9
2	>99.9a
3	>99.9a
4	>99.9a
5	>99.9a
6	>99.9
7	>99.9
8	>99.9a
9	>99.9a
10	>99.9a
11	>99.9
12	>99.9
13	>99.9
14	>99.9
15	>99.9a
16	>99.9
17	>99.9a
18	>99.9a
19	>99.9a
20	>99.9a

ASTM F2100 Requirements for Level 2 pass	>98

Note: There were no detected colonies on any of the Andersen sampler plates for this test article.

Conclusions: The filter media exceeded the ASTM F2100 Level 2 bacterial filtration efficiency requirement of >98%.

Table S5. Viral filtration efficiency of PLA NF filter media.

Test Article Number	Percent VFE (%)
1	>99.9
2	>99.9a
3	>99.9a
4	>99.9a
5	>99.9
6	>99.9a
7	>99.9
8	>99.9
9	>99.9a
10	>99.9a

Conclusions: The filter media showed a >99.9 viral filtration efficiency.

3.1.2. ASTM F3502 - Standard Specification for Barrier Face Coverings

Table S6. Filtration efficiency of PLA NF filter when challenge with 0.3 μm NaCl particles following ASTM F3502 test method, certified by Nelson Lab.

Test Article Number	Airflow Resistance (mm H2O)	Airflow Resistance (Pa)	Filtration Efficiency (%)
1	8.8	86.0	85.9
2	9.9	96.9	93.5
3	9.9	97.2	89.7
4	12.2	120.0	99.3
5	11.5	113.2	97.6
6	10.2	100.4	87.7
7	11.0	108.4	95.0
8	12.2	119.7	93.4
9	9.6	93.8	93.0
10	13.1	128.5	97.1

Conclusions: The filter media exceeded the ASTM F3502 Level 2 particle filtration efficiency requirements of ≥50 % as well as meeting the Level 1 airflow resistance requirements of ≤15 mm H_2O.

Table S7. Filtration efficiency of PLA NF filter when challenge with 0.3 μm NaCl particles following ASTM F3502 test method after 10 laundering cycles, certified by Nelson Lab.

Test Article Number	Airflow Resistance (mm H2O)	Airflow Resistance (Pa)	Filtration Efficiency (%)
1	10.6	42.2	57.8
2	11.3	38.2	61.8
3	12.1	35.0	65.0
4	10.1	41.6	58.4
5	8.0	61.1	38.9
6	12.5	34.4	65.6
7	10.4	40.5	59.5

8	10.9	39.5	60.5
10	12.4	36.3	63.7

3.1.3. NIOSH - 42 CFR Part 84 - Respiratory Protective Devices

Table S8. Filtration efficiency of PLA NF filter when challenge with 0.3 µm NaCl particles following NIOSH – 42CFR84 test method, certified by Nelson Lab.

Test Article Number	Inhalation Resistance (Pa)	Exhalation Resistance (Pa)	Maximum 0.3 µm Particle Penetration (%)	0.3 µm Particle Filtration Efficiency (%)
1	206	144	0.0	100.0
2	263	184	0.0	100.0
3	228	157	0.0	100.0
4	188	129	2.1	98.0
5	250	174	0.0	100.0
6	213	153	3.3	96.7
7	139	94	1.9	98.1
8	213	147	0.0	100.0
9	291	203	3.6	96.4
10	238	138	0.0	100.0
11	264	184	1.0	99.0
12	290	201	0.0	100.0
13	250	174	0.4	99.6
14	269	188	2.5	97.5
15	273	190	0.0	100.0
16	257	178	0.0	100.0
17	295	204	0.0	100.0
18	180	124	4.5	95.5
NIOSH N95 Requirements	<314Pa	<245Pa	≤5	≥95
